# Supplementary material for: Predictors of mortality within the first year of initiating antiretroviral therapy in urban and rural Kenya: A prospective cohort study
Source: PLoS One. 2019 Oct 4;14(10):e0223411. doi: 10.1371/journal.pone.0223411 (PMC6777822; doi:10.1371/journal.pone.0223411)
Supplement: S1 Table — Details on ART initiation status, gender, age, body mass index, CD4 count, pre-treatment drug resistance status, number of days from study enrollment to death, time from ART initiation to death, and summary of cause of death and/or symptoms at time of death when available by location: A) Nairobi and B) Maseno. (DOCX) [file pone.0223411.s001.docx]

**S1 Table. List of baseline correlates and summary of cause/symptoms at the time of death (n=81)**

1. Nairobi (n=52)

| ART  Initiated | Gender | Age | BMI  (m/kg^2^) | CD4 Count  (cells/µL) | PDR | Days from  Enrollment  to Death | Days from  ART start  to Death | Simple Summary of Cause/  Symptoms at Death |
| --- | --- | --- | --- | --- | --- | --- | --- | --- |
| No | Female | 27 | 16.5 | 52 | unknown | 20 |  | Severe anemia |
| No | Female | 27 | 21.9 | 4 | wildtype | 66 |  | General body weakness, difficulty breathing, sweating profusely |
| No | Female | 30 | 19.1 | 14 | wildtype | 31 |  | TB |
| No | Female | 35 | 19.7 | 6 | wildtype | 4 |  | Kidney disease, tuberculosis (TB) |
| No | Female | 35 | 11.1 |  | wildtype | 22 |  | Severe anemia, esophageal candidiasis, lower respiratory tract infection, genital ulcer disease |
| No | Female | 38 | 21.6 | 43 | wildtype | 29 |  | TB |
| No | Female | 39 | 33.2 | 7 | wildtype | 105 |  | Collapsed and died |
| No | Female | 44 | unknown | 135 | wildtype | 36 |  | TB, difficulty breathing, chest pain, cough, swollen left leg and arm |
| No | Female | 48 | 18.2 | 214 | wildtype | 231 |  | Cervical cancer |
| No | Male | 30 | 17.8 | 471 | wildtype | 6 |  | Collapsed and died |
| No | Male | 33 | 16.9 | 81 | wildtype | 15 |  | Diarrhea & vomiting postprandial associated with dysphagia, sick looking, pale, oral thrush |
| No | Male | 36 | 19.3 | 4 | wildtype | 26 |  | Difficulty breathing |
| No | Male | 37 | 23.2 | 31 | wildtype | 18 |  | Anemia & pneumonia |
| No | Male | 38 | 19.8 | 196 | wildtype | 400 |  | Unknown (weakness) |
| No | Male | 40 | 19.4 | 14 | wildtype | 179 |  | Unknown |
| No | Male | 47 | 22.5 | 24 | wildtype | 3 |  | TB, headache & generalized body aches |
| No | Male | 52 | 15.2 | 176 | wildtype | 20 |  | TB |
| Yes | Female | 29 | 18.7 | 4 | wildtype | 52 | 38 | Body weakness, anemia, headache, chronic diarrhea, & vomiting |
| Yes | Female | 30 | 23.9 | 473 | wildtype | 203 | 189 | Kidney disease (anemia and dialysis) |
| Yes | Female | 31 | 13.6 | 56 | 10-100% | 394 | 375 | Unknown |
| Yes | Female | 34 | 18.7 |  | 10-100% | 192 | 157 | Diarrhea, general body malaise, vomiting, weight loss, reduced appetite, cachexic & dehydrated |
| Yes | Female | 37 | 22.5 | 20 | wildtype | 40 | 20 | Anemia & oral candidiasis & swelling/weakness of lower limbs |
| Yes | Female | 37 | 28.4 | 68 | wildtype | 291 | 271 | Postprandial vomiting, epigastric abdominal pains, anemia |
| Yes | Female | 39 | 22.1 | 21 | 10-100% | 32 | 18 | Headache, general body malaise, diarrhea, vomiting, dehydration |
| Yes | Female | 41 | 15.9 | 118 | wildtype | 74 | 38 | General body weakness, body itchiness, rash, & wounds |
| Yes | Female | 44 | 16.5 | 44 | wildtype | 17 | 6 | Chest congestion, difficulty breathing, underweight, anemia |
| Yes | Female | 45 | 20.1 | 57 | wildtype | 93 | 81 | TB |
| Yes | Female | 49 |  | 64 | wildtype | 72 | 57 | Pneumonia |
| Yes | Female | 52 | 17 | 22 | 2-9% | 288 | 274 | Cancer: Kaposi’s Sarcoma (possibly) |
| Yes | Female | 54 | 26.4 | 321 | wildtype | 290 | 270 | Kidney disease |
| Yes | Female | 57 | 26.6 | 292 | wildtype | 382 | 365 | TB |
| Yes | Female | 61 | 15.6 | 76 | wildtype | 78 | 64 | Cryptococcal meningitis, oral candidiasis, & moderate anemia. |
| Yes | Female | 62 | 20.3 | 80 | wildtype | 105 | 89 | Unknown (general illness) |
| Yes | Male | 32 | 16.6 | 77 | wildtype | 195 | 44 | Meningitis (severe headache/paralysis on one side) |
| Yes | Male | 33 | 17 | 318 | wildtype | 77 | 63 | Edema of lower limbs, difficulty breathing, & unconscious episodes |
| Yes | Male | 33 | 18.1 | 44 | wildtype | 21 | 7 | General malaise |
| Yes | Male | 34 | 19.8 | 42 | wildtype | 127 | 78 | Cancer: Kaposi’s Sarcoma, possible metastasis to the lungs & pneumonia (PCP) |
| Yes | Male | 35 | unknown | 153 | wildtype | 143 | 96 | Leg weakness & resting tremors; paralysis of one side |
| Yes | Male | 35 | 18.8 | 142 | wildtype | 88 | 74 | Yellowing eyes & urine, general body malaise, fever |
| Yes | Male | 37 | 27.4 | 136 | wildtype | 129 | 107 | Pneumonia (PCP) & TB |
| Yes | Male | 37 | 24.2 | 246 | 10-100% | 444 | 431 | Cancer: Kaposi’s Sarcoma, metastasis to chest |
| Yes | Male | 39 | 18.3 | 14 | wildtype | 115 | 101 | Cancer: Kaposi’s Sarcoma |
| Yes | Male | 40 | 25.2 | 150 | wildtype | 70 | 50 | Cough, severe vomiting (blood stained) |
| Yes | Male | 42 | 21.3 | 114 | wildtype | 21 | 5 | TB, acute renal illness, & UTI |
| Yes | Male | 47 | 27.7 | 101 | wildtype | 431 | 411 | Ruptured pancreas |
| Yes | Male | 48 | 20.3 | 10 | wildtype | 28 | 11 | Poor appetite, weight loss, & anemia |
| Yes | Male | 50 | 20.2 | 543 | wildtype | 246 | 228 | Collapsed and died |
| Yes | Male | 50 | 19 | 21 | wildtype | 200 | 170 | TB |
| Yes | Male | 51 | 15.5 | 32 | 10-100% | 44 | 30 | Diarrhea, weakness |
| Yes | Male | 52 | 17 | 134 | wildtype | 89 | 73 | Severe pneumonia & severe anemia |
| Yes | Male | 58 | 20.8 | 24 | wildtype | 69 | 9 | Lower abdominal pains, difficulty breathing, & low blood glucose |
| Yes | Male | 60 | 24.2 | 1560 | wildtype | 39 | 15 | Diabetes |

1. Maseno (n=29)

| ART  Initiated | Gender | Age | BMI  (m/kg^2^) | CD4 Count  (cells/µL) | PDR | Days from  Enrollment  to Death | Days from  ART start  to Death | Simple Summary of Cause/  Symptoms at Death |
| --- | --- | --- | --- | --- | --- | --- | --- | --- |
| No | Female | 34 | 14.7 | unknown | wildtype | 10 |  | Persistent diarrhea, difficulty breathing, & severe chest pain |
| No | Male | 29 | 18.8 | 80 | wildtype | 31 |  | Difficulty breathing, severe chest pain, general body malaise |
| No | Male | 56 | 17.2 | 29 | wildtype | 30 |  | Dizziness, generalized malaise, anorexia, severe dehydration, immuno-suppression, & esophageal candidiasis |
| Yes | Female | 23 | 13.9 | 300 | wildtype | 409 | 395 | Wasted & anorexic, vomiting, bilateral edema, yellow eyes, oral sores, skin excoriation, very weak, inability to walk unaided |
| Yes | Female | 28 | 14.1 | 26 | wildtype | 365 | 351 | Emaciated, vomiting, diarrhea & had yellow eyes |
| Yes | Female | 29 | 18 | 98 | 2-9% | 56 | 42 | Psychological symptoms, collapsed and died, possible TB meningitis |
| Yes | Female | 29 | 17.4 | 263 | 10-100% | 82 | 68 | Vomiting, anorexic, diarrhea, & cough |
| Yes | Female | 30 | 17.4 | 116 | wildtype | 54 | 26 | Severe cough (2 weeks), chest congestion, & difficulty breathing |
| Yes | Female | 30 | 25.1 | 4 | 2-9% | 351 | 337 | Cryptococcal meningitis |
| Yes | Female | 32 | 17.1 | 151 | wildtype | 37 | 16 | Vomiting, appetite loss, immobility, severe dehydration (gastroenteritis) |
| Yes | Female | 37 | 18.3 | 23 | 2-9% | 96 | 82 | Anorexia, general malaise, weight loss, bilateral lower limb swelling, & inability to walk |
| Yes | Female | 39 | 16.2 | 133 | wildtype | 65 | 30 | Severe diarrhea, vomiting, lower limb edema |
| Yes | Female | 39 | 16.4 | 39 | wildtype | 29 | 15 | Oral candidiasis, gastroenteritis/dehydration, anemia |
| Yes | Female | 46 | 19.8 | 243 | wildtype | 29 | 15 | Diarrhea & difficulty breathing |
| Yes | Female | 47 | 18.3 | 51 | wildtype | 17 | 6 | Anorexia, general body malaise |
| Yes | Female | 48 | 16.8 | 45 | wildtype | 42 | 16 | Severe headache, vomiting, dehydration, gasping |
| Yes | Female | 54 | 21.9 | 6 | wildtype | 51 | 30 | Anemia |
| Yes | Female | 56 | 20.3 | 341 | wildtype | 195 | 181 | Unknown |
| Yes | Female | 56 | 16.2 | 38 | wildtype | 52 | 38 | Vomiting blood, blood stained diarrhea, both feet became swollen, peptic ulcer disease |
| Yes | Male | 25 | 14.9 | 5 | wildtype | 231 | 217 | Diarrhea, vomiting, general malaise |
| Yes | Male | 25 | 15.9 | 33 | wildtype | 456 | 442 | TB, loss of appetite, coughing, weakness |
| Yes | Male | 32 | 16.9 | 20 | 10-100% | 119 | 105 | Unknown |
| Yes | Male | 34 | 19.8 | 69 | wildtype | 85 | 62 | TB, liver failure |
| Yes | Male | 39 | 18.2 | 135 | wildtype | 469 | 455 | Abdomen swelling, low appetite, difficulty breathing, general weakness, pedal edema |
| Yes | Male | 47 | 19.3 | 95 | wildtype | 101 | 87 | Vomiting & headache, TB |
| Yes | Male | 51 | 14.5 | 32 | 10-100% | 84 | 71 | Severe diarrhea |
| Yes | Male | 62 | 18.2 | 42 | 10-100% | 39 | 24 | Dyspnea, chest pain, & herpes zoster |
| Yes | Male | 71 | 17.5 | 48 | wildtype | 198 | 182 | Cancer: throat, swollen mouth, aphasia, & anorexia |
| Yes | Male | 85 | 17.1 | 47 | wildtype | 166 | 152 | Pneumonia |
